# TGV: suite of tools to visualize transmission graphs

**DOI:** 10.1093/nargab/lqae158

**Published:** 2024-12-05

**Authors:** Jody E Phelan, Fatima Niazi, Linfeng Wang, Gabrielle C Ngwana-Joseph, Benjamin Sobkowiak, Ted Cohen, Susana Campino, Taane G Clark

**Affiliations:** Department of Infection Biology, London School of Hygiene and Tropical Medicine, Keppel St, London WC1E 7HT, UK; University College London Hospitals NHS Foundation Trust, Eastman Dental Hospital, Huntley Street, London, WC1E 6DG, UK; Department of Infection Biology, London School of Hygiene and Tropical Medicine, Keppel St, London WC1E 7HT, UK; Department of Infection Biology, London School of Hygiene and Tropical Medicine, Keppel St, London WC1E 7HT, UK; Department of Epidemiology of Microbial Disease, Yale School of Public Health, 60 College St, New Haven, CT 06510, USA; Department of Epidemiology of Microbial Disease, Yale School of Public Health, 60 College St, New Haven, CT 06510, USA; Department of Infection Biology, London School of Hygiene and Tropical Medicine, Keppel St, London WC1E 7HT, UK; Department of Infection Biology, London School of Hygiene and Tropical Medicine, Keppel St, London WC1E 7HT, UK; Faculty of Epidemiology and Population Health, London School of Hygiene and Tropical Medicine, Keppel St, London WC1E 7HT, UK

## Abstract

Graph structures are often used to visualize transmission networks generated using genomic epidemiological methods. However, tools to interactively visualize these graphs do not exist. A browser-based tool allowing users to load and interactively visualize transmission graphs was developed in JavaScript. Associated metadata can be loaded and used to annotate and filter the nodes and edges of transmission networks. The tool is available at jodyphelan.github.io/tgv.

## Graphical Abstract

**Graphical Abstract:**
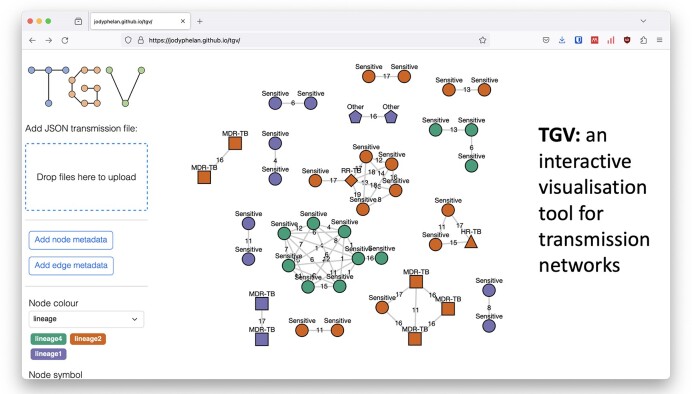

## Introduction

Tracking outbreaks of infectious diseases has traditionally relied on mathematical modelling approaches to aggregate estimates of transmission. Genomics has ushered in a revolutionary era in tracking the transmission of infectious agents. Using next-generation sequencing, the genomes of bacteria and viruses can be characterized and compared, finding differences between isolates and enabling the fine-scale mapping of transmission chains or networks. Exploring these networks can help us to better understand pathogen transmission dynamics to provide insights into biological, social and demographic factors that influence the spread of disease, particularly when coupled with linked epidemiological data.

Simple approaches to find potential transmission events typically include estimating the number of mutation [e.g. single-nucleotide polymorphism (SNP)] differences between samples and applying a species- and population-specific difference cut-off to classify them as related. This approach has been applied extensively to identify the transmission of *Mycobacterium tuberculosis*, the cause of tuberculosis disease (TB) (1). The resulting ‘relatedness’ network forms a graph structure where the nodes are sequences and the edges linking the nodes represent a potential transmission link with a value equal to the SNP distance. More sophisticated approaches incorporate important parameters such as sampling time, population size and geographic location, leading to a graph where the edges have a probability value, which indicates how probable the two sequences are linked by a recent transmission. These graphs can also be directional, where a probability is given for a specific direction of transmission.

Phylogenetic trees are often used to find closely related sequences indicated by the short branch lengths separating any two sequences. While useful, they lack the ability to characterize fine-scale transmission events. One advantage of using phylogenetics has been the ease at which it is possible to visualize genomics relatedness in large datasets. Most phylogenetic tools output a tree in Newick (2) or Nexus (3) format and these can be easily visualized by a variety of interactive tools such as iTOL (4) and phandango (5). Additional metadata can also be layered onto these trees to produce an informative graphic that can convey multi-dimensional information about strain relatedness. The graphical and interactive aspect of these tools greatly contribute towards their popularity, allowing the user to alter the presentation of the tree to best present the message they are trying to convey.

A recent review of data visualization tools used to investigate healthcare infections found that transmission networks were consistently utilized across the literature (6). These networks were often supplemented with patient characteristic data to provide a more comprehensive analysis (6). While tools to visualize transmission networks exist, they are mostly used through programming tools such as rgraphviz for R. This visualization process requires a user to know advanced programming, thereby representing a major barrier for non-bioinformaticians to view and analyse the results from these analyses. Additionally, the non-interactive nature of these tools means that the visualization of outputs requires several iterations to get right. As such, the value of interactive tools in this field has been recognized, with recent publication of tools such as HAIviz (7) that have network visualization capabilities. A potential reason for the lack of tools in this area is the non-standardization of outputs from different analysis software. Newick and Nexus have become the standard formats, and though this has enabled the development of interoperable phylogenetic tools, this standardization has been lacking for transmission graphs. Transmission tools generally output SNP distances or transmission probabilities in a matrix format. While this leads to fast data processing (e.g. look-ups), it has a large disk usage footprint that could be reduced for sparse matrices, where there are few linked samples. This format also makes it difficult to store more than one attribute between samples.

JavaScript object notation (JSON) format is a text-based format that is human-readable and flexible for storing unstructured data. Several, specialized formats based on JSON have been used in a variety of areas. For example, the GeoJSON format has become an industry standard for storing spatial information, and schemas have been created to store phylogenetic and sequence data. The format also has an advantage that it maps straight to the Python dictionary data structure and JavaScript objects, making the parsing and loading of data easy.

To solve the issues outlined above, we present a schema for the use of JSON to store transmission information, a command-line tool to import, convert and manipulate outputs from existing transmission inference tools, and a web-based graphical tool to enable easy visualization of networks.

## Materials and methods

### Storage format

A JSON schema, termed trjson, was designed to allow for attributes to be stored for both the samples (nodes) and the links between them (edges). Nodes and edges are stored as objects in dedicated arrays. Nodes are required to have a unique ID attribute and edges are required to have source and target attributes referencing the node IDs. Additional attributes can then be flexibly defined as strings, floating point numbers or integers. Definitions for each attribute can also be stored.

### Data conversion and manipulation

A command-line tool was developed to convert outputs from transmission inference tools into trjson format. The tool, named tgtools, was developed in the Python language and is hosted at https://github.com/jodyphelan/tgtools. Tgtools also allows for the manipulation of graphs, including the extraction of subgraphs, refining edges based on new attribute cut-offs and the merging of additional metadata on nodes or edges.

### Network display

Finally, a web-based graphical tool named Transmission Graph Viewer (TGV) was developed in HTML, CSS and JavaScript using Cytoscape.js to render graphs and the bootstrap HTML framework for styling. The tool is hosted on GitHub and can be accessed at https://jodyphelan.github.io/tgv/. A transmission graph is loaded by dropping a file into an uploader, which triggers the graph to render. The graph layout can then be manually altered by dragging the nodes around. All data loading is performed within the browser, meaning it never leaves the computer of the user, thus maximizing security and its protection. Directed graphs can have different probabilities between two nodes, and an arrow is displayed from the source. Undirected graphs will simply have one edge between pairs of nodes.

### Annotation

**Figure 1.**
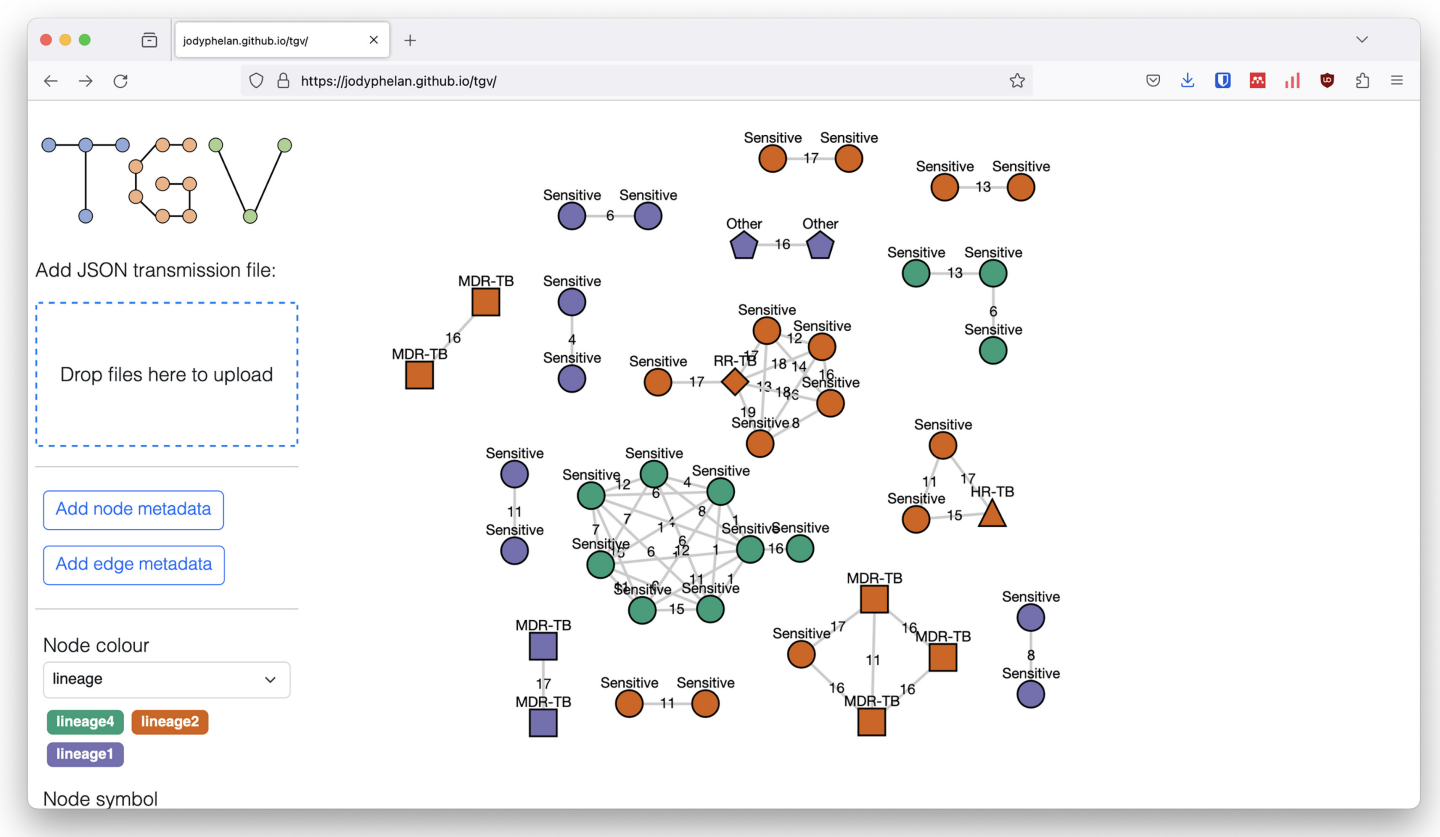
Visualization of graphs and annotation. The transmission network can be annotated by labelling nodes and edges and changing the shape and colour of the nodes. This graph has been generated from an *M. tuberculosis* SNP distance matrix and has been annotated with a drug resistance phenotype, where HR-TB refers to isoniazid resistance and MDR-TB is resistance to isoniazid and rifampicin drugs. This represents an undirected graph as there is only one distance value estimated between each pair.

Additional metadata can be displayed on the graph in the form of text labels and changing the node shapes and colours (Figure 1). Labelling the edges is often desirable to show the probabilities between pairs of nodes. By default, for each attribute with up to 12 values, a qualitative colour scheme will be selected to maximize divergence between categories. Colour palettes can then be altered with the user reassigning colours for each value.

### Live manipulation

**Figure 2.**
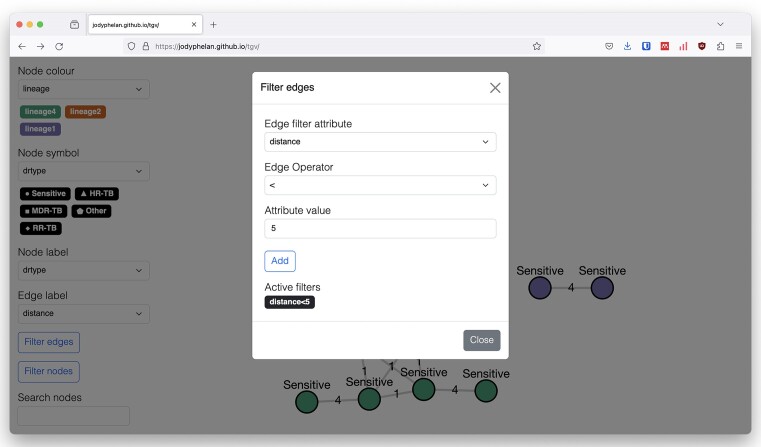
Real-time modification of graphs. Graph manipulation can be performed by applying Boolean statements to node or edge attributes. In this example, the stringency of the transmission linkage is made stricter by applying a lower distance threshold.

The graph structure can be altered in memory with Boolean statements that can be applied to the node and edge attributes. For example, this could allow the user to refine the connectivity between the nodes using stricter probability cut-offs or removing nodes from the visualization based on node attributes such as geographical location (Figure 2).

### Export

It is also possible to freely zoom in and out of sections of the graph and use a text-based search on the node IDs to locate nodes of interest. Subgraphs can be selected with a click-and-drag interface after which the names of the nodes can be copied to the clipboard. The graphic can also be exported to a high-resolution PNG file for the generation of publication-ready figures.

## Results

### Application to *M. tuberculosis* sequence data and transmission analysis

To demonstrate the use of this tool, we analysed a publicly available dataset of *M. tuberculosis* whole genome sequence data collected from all TB culture-positive individuals from the Republic of Moldova between January 2018 and December 2019 (1). High-quality short-read sequence public datasets were available for 2770 culture-positive TB individuals. Demographic, epidemiological and disease data, including age, sex, location and smear status, were collected for everyone.

Raw sequencing reads were downloaded from the European Nucleotide Archive (ENA; accession: PRJNA736718) and aligned to the H37Rv reference strain (NCBI accession no. NC_000962.3) using bwa-mem (8), with sorted binary alignment files created using samtools software (v.1.10) (9). TB lineage and *in silico* antimicrobial resistance profiles were predicted using TB-Profiler software (10). Variant calling was carried out with GATK (v. 4.1) (11) to identify SNPs, with low-quality variants removed and mixed calls assigned as the majority allele if >80% of reads were called as the nucleotide. SNPs found in *pe*/*ppe* genes, repetitive regions and at known antimicrobial resistance conferring sites were also removed. High-likelihood mixed infection samples identified by MixInfect (12) were also removed from further analysis, resulting in a final dataset consisting of 1834 individuals.

**Figure 3.**
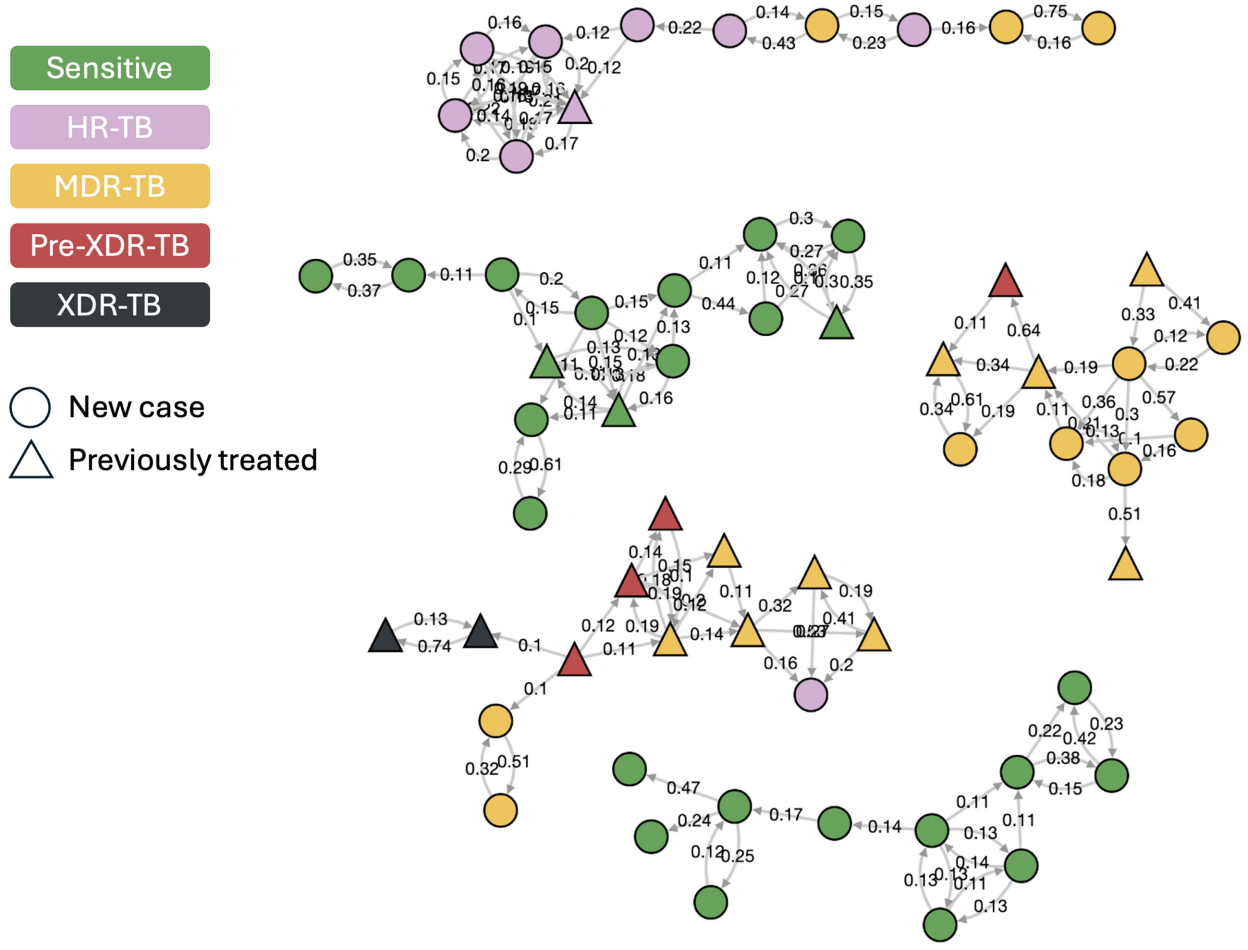
Transmission of *M. tuberculosis* using whole genome sequence data. Transmission clusters of *M. tuberculosis* were generated from whole genome sequence data generated from Moldovan clinical isolates using TransPhylo. The graph shows the potential transmission of MDR-TB and XDR-TB. This graph is directional as probabilities between each pair are estimated for both directions.

The probability of direct transmission between individuals with TB in the population was inferred using the multi-input tree implementation of TransPhylo (13,14). First, broad clusters were identified by linking sequences in groups with a pairwise distance of ≤50 SNPs. Next, a timed phylogeny was built for each of these clusters using BEAST2 (15) with the HKY nucleotide substitution model, a strict clock model and constant population size. Models were run for 2.5 × 10^8^ Markov chain Monte Carlo (MCMC) iterations or until convergence and 50 trees for each cluster were drawn from the posterior distributions, after a 20% burn-in, to be used as input for TransPhylo. The TransPhylo infer_multittree_share_param function was used to reconstruct transmission from the phylogenies using a prior gamma generation time (*k =*1.3, *θ* = 3.33) and sampling time (*k =*1.1, *θ* = 2.75) distribution, a beta sampling density distribution (*α* = 20, *β* = 8) and a fixed within-host coalescent rate of 100/365. Models were run for 10^5^ MCMC iterations and prior parameters shared across the 50 independent runs per cluster. Finally, we used the computeMatWIW function to calculate the posterior probability of directed transmission between individuals in each cluster and imputed a zero probability of transmission between those not in the same cluster. Tgtools was used to convert the probability matrix into trjson format. The transmission clusters were visualized and annotated with TGV (Figure 3). The output highlights the transmission of MDR-TB and extensively drug-resistant TB (XDR-TB) within the largest five clusters of *M. tuberculosis*. Files for this example can be found at https://github.com/jodyphelan/tgtools/tree/main/examples/Moldova_Tb.

### Application to *Plasmodium vivax* identity by descent data to infer malaria transmission

**Figure 4.**
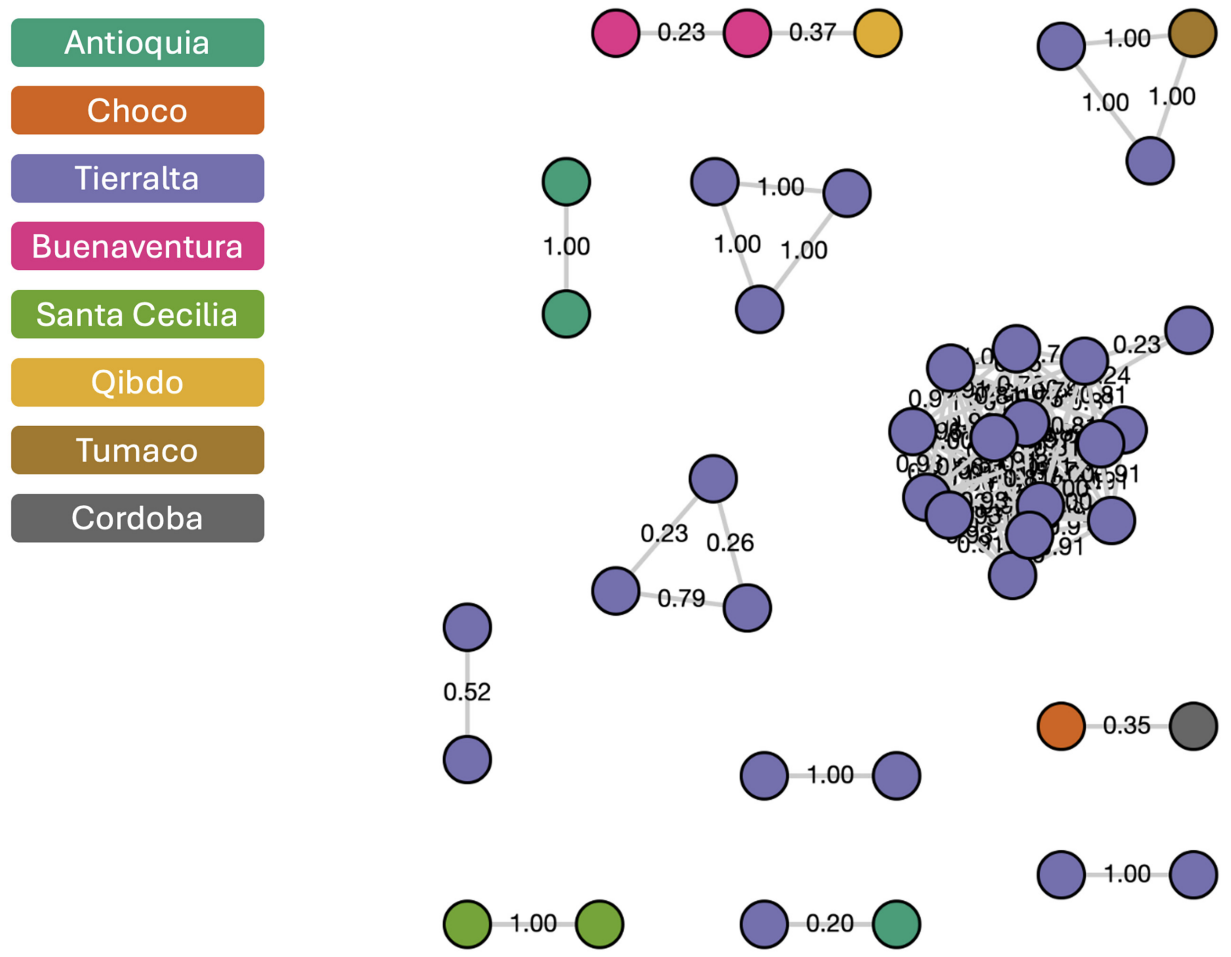
IBD networks visualized using TGV. IBD networks were generated from whole genome sequence data generated from Colombian clinical *P. vivax* isolates using hmmIBD. The network reveals the presence of highly related strains, indicative of transmission, across different geographical regions.

We also demonstrate the use of TGV with genetic relatedness graphs frequently used to look at malaria parasite transmission (16). Detecting segments of shared ancestry [identity by descent (IBD)] is a fundamental estimation of genetic relatedness. Hidden Markov models provide a framework to infer pairwise IBD segments from genetic data and are commonly used in studies of malaria transmission dynamics and relatedness. Using a matrix of 112 816 high-quality genome-wide biallelic SNPs, hmmIBD (16) was applied to 54 monoclonal *P. vivax* isolates of Colombian origin (17,18). For each pair of isolates, the fraction of sites called identical was estimated with the Viterbi algorithm. Values are reported between 0 and 1, where clonal isolate pairs have a relatedness estimate of 1 and completely unrelated isolate pairs have an estimate of 0. Tgtools was used to convert the IBD matrix into trjson format. Highly related isolate pairs were classified using an arbitrary threshold of 0.2. The IBD graph was visualized with TGV (Figure 4) and shows that the majority of the clustered isolates are from the Tierralta region of Colombia. Files used for this example are found at https://github.com/jodyphelan/tgtools/tree/main/examples/Colombia_Pv.

## Discussion

We have developed a set of tools for the import and interactive visualization of transmission graphs. Though the tool was designed to visualize transmission links, it can be used to visualize any relatedness or linkage network. Graphs are stored via a specific subset of JSON format and can be converted from a number of commonly used formats such as an SNP distance matrix. TGV was developed with flexibility in mind, allowing users to layer on annotations that improve the amount of information relayed through the visualization, thus improving the interpretability and application of transmission analyses.

## Data Availability

The web server is available at https://jodyphelan.github.io/tgv/. All code is available for TGV at https://github.com/jodyphelan/tgv (doi: https://doi.org/10.6084/m9.figshare.27330822) and for tgtools at https://github.com/jodyphelan/tgtools (https://doi.org/10.6084/m9.figshare.27330819). *Mycobacterium tuberculosis* sequence data were downloaded from the ENA under the project accession PRJNA736718. *Plasmodium vivax* sequence data were downloaded from the ENA under the project accessions PRJEB44419, PRJNA240438 and PRJEB2140.
